# Adolescent ethanol experience alters immediate and long-term behavioral responses to ethanol odor in observer and demonstrator rats

**DOI:** 10.1186/1744-9081-5-23

**Published:** 2009-06-04

**Authors:** Amber M Eade, Steven L Youngentob

**Affiliations:** 1 Department of Neuroscience and Physiology, State University of New York Upstate Medical University, Syracuse, New York, USA; 2 State University of New York Developmental Exposure Alcohol Research Center, Syracuse & Binghamton, New York, USA

## Abstract

**Background:**

The social transmission of food preference paradigm centers on the finding that observers obtain dietary information through olfactory cues on the breath of a demonstrator peer that has ingested a novel substance. This phenomenon plays a role in ethanol acceptability. Historically, studies using this technique have focused on observer animals in order to study the social transmission process. With respect to ethanol, studies of acute intoxication have shown that the pharmacologic properties of ethanol and hematogenic olfaction can influence the subsequent ethanol odor-mediated responses of the intoxicated animals. These acute studies, however, demonstrate odor aversion. The present study compared the effect of adolescent ethanol exposure, via the social transmission paradigm, on the behavioral response to ethanol odor in both observer and demonstrator animals in adolescence (postnatal day (P) 37) and the persistence of these effects into adulthood (P90).

**Methods:**

Beginning on P29, naïve rats received four ethanol or water exposures: one every 48 hours through either direct intragastric infusion or social interaction with an infused peer. The reflexive sniffing response of observers and demonstrators to ethanol odor was tested at P37 or P90 using whole-body plethysmography.

**Results:**

The behavioral response of adolescent ethanol observers and demonstrators significantly differed between themselves and from their respective water controls. Ethanol and water observers both displayed a greater response to ethanol odor than their respective demonstrator counterparts. Compared to controls, both modes of ethanol exposure produced similar magnitudes of enhancement. At P90, both forms of exposure displayed similar responses to ethanol odor and similar magnitudes of enhancement. Only demonstrators displayed equivalent enhanced responses in both sexes.

**Conclusion:**

In contrast to previous studies showing odor aversion following acute ethanol intoxication, within the context of the social transmission paradigm, adolescent demonstrators like observers showed an enhanced behavioral response to ethanol odor. The differential enhanced odor response between observers and demonstrators, despite similar net enhancements relative to controls, suggests the presence of a stress effect from the infusion technique. This finding contrasts previous suggestions that intragastric infusions create minimal stress: an important consideration when conducting ethanol research. This stress effect appears to ameliorate by adulthood.

## Background

The ability for food preferences in rats to be influenced through social interactions centers on the observation that rodents can obtain new appetitive dietary information, at least in part, by interacting with a peer that has ingested a novel substance. It is specifically the olfactory cues perceived on the breath of an animal (i.e. demonstrator) that ingested the "novel food" that have been found to impact the acceptability of the substance by a conspecific (i.e.observer) [1-3]. This general phenomenon has been shown to play a role in ethanol acceptability. Several studies have demonstrated that both naïve infant and adolescent observer rats will increase their preference for ethanol odor, as well as manifest enhanced ethanol intake, as a consequence of interaction with a peer that was administered ethanol [e.g., [4-9]]. Further, there is evidence of an interaction between pre- and post-natal exposure on ethanol intake [9,10]. In this later regard, recent work has demonstrated the important contribution of olfaction, per se, to this interactive effect [4]. That is, experiencing ethanol odor in adolescence, as an observer, not only enhances the olfactory behavioral response to ethanol odor but also augments the known behavioral alterations due to prior fetal exposure with the drug.

Given the important role that the social transmission of odor information plays in ethanol odor and intake preference it is interesting that previous work has by in large focused exclusively on the observer animals. Within the context of the social transmission paradigm it is likely that either the pharmacologic properties of ethanol alone or in paired concert with hematogenic olfaction [11-13] would also significantly impact the subsequent odor-mediated responses of the demonstrator animals to ethanol. To be sure, it has been implied from acute alcohol intoxication studies that pre-weanling animals acquire associations between ethanol's orosensory cues arising from non-metabolic routes of elimination and its pharmacologic properties [14-16]. Interestingly, however, these studies demonstrated that acute intoxication via intragastric (i.g.) infusion yielded ethanol odor aversion. As such, within the context of the social transmission paradigm, observer/demonstrator affects may differ.

Human adolescents may encounter ethanol either directly, through personal ingestion, or indirectly, through interacting with others who have ingested the drug. In this respect, the social transmission paradigm permits the simultaneous assessment of ethanol-related odor information through two potentially relevant forms of human interaction [17] that can be termed direct (i.e. systemic experience with ethanol) and indirect (i.e. experience via social interaction with an exposed peer). Although both forms of exposure have individually been shown to produce altered odor responses (albeit in opposite directions), a direct comparison of these effects, particularly in adolescent animals, has not been subjected to published investigation. Therefore, using the social interaction paradigm we compared the behavioral response of adolescent (postnatal day (P) 37) observer and demonstrator animals to ethanol odor, following adolescent exposure to the drug. Moreover, we compared whether both forms of ethanol exposure yield persistence of the olfactory effect into adulthood (P90).

## Methods

### Subjects

A total of 96 naive Long-Evans Hooded rats, the progeny of 12 dams, were utilized in this study. Pregnant Long-Evans female rats (Harlan-Sprague Dawley, Indianapolis, IN) were allowed ad-libitum access to a liquid diet (L10252, Research Diets, NJ) and water throughout gestation. Litters were sexed and culled to 10 pups each on the morning of P2, ensuring that litters contained no fewer than 4 males and 4 females. Animals were housed at SUNY Upstate Medical University in a temperature and humidity controlled environment with a fixed 12-hour light/dark cycle. All procedures were performed in accordance with the guidelines set by the SUNY Upstate Medical University Institutional Animal Care and Use Committee.

### Adolescent ethanol exposure

Adolescent exposure was accomplished using a social transmission of food odor paradigm in which a substance is given to one animal (demonstrator) and information about that substance is transmitted, specifically through the olfactory system, to a peer (observer) during a period of social interaction [1-3].

At weaning (P21), the eight pups from each litter were randomly allocated into cages of 2 same sex siblings that remained housed together for the remainder of the experiment [4]. Within each pair, one animal was randomly designated as the demonstrator and the other as the observer. One same sex pair from each litter was randomly selected to receive experience with water in adolescence while the other pair experienced ethanol. Thus, both animals within a same sex cage would experience the same substance: one through direct intragastric infusion, i.e. the demonstrator animal, and the other through social interaction with the infused peer, namely, the observer.

Experience with either ethanol or water occurred 4 times, 48 hours apart, beginning on P29 (that is, on P29, P31, P33, & P35). On each day of exposure, pairs were separated for 1 hr prior to social interaction by removing the demonstrator from the home cage. Thirty minutes into the separation period, demonstrators were i.g. infused with either a sub-narcoleptic dose of 1.5 g/kg ethanol (a dose that has been found to increase odor preference and consumption of ethanol by naïve adolescent observers without inhibiting social activity) or an equivalent volume of tap water [6,8]. Intragastric infusion occurred as previously described [4,9,17]. Thirty minutes after the infusion the demonstrator was returned to its home cage for 30 minutes of social interaction with the observer. Animals were again separated for 4 hours after the social interaction period to permit ethanol to clear the ethanol demonstrator's system prior to being housed with the observer sibling overnight.

### Stimulus-induced reflexive sniffing behavior

The present study took advantage of a technique that utilizes the monitoring of stimulus-induced sniffing as a method for the quantification of the attentiveness/responsivity to odorant stimuli [4,18-21]. Briefly, changes in stimulus-induced sniffing in response to ethanol odor were monitored according to previously established procedures using whole-body plethysmography (*ibid*). The odor-testing chamber permitted rapid stimulus onset and clean out. Odor stimuli were generated using a standard flow-dilution olfactometer and electronic mass flow controllers (Teledyne Co, Hampton, Virginia, USA). All stimulus generation and presentation, as well as data collection were computer controlled.

Each experimental animal was tested once either during late adolescence (P37), 48 hours after the final ethanol social interaction experience, or in adulthood (P90) [22]. Following a 40 trial air-only habituation period, air and ethanol odor trials were presented in blocks of 10 air and 10 odor stimuli using a fixed 6s inter-trial interval schedule. Odor stimuli consisted of an ascending series of five concentrations of ethanol odor (0.313%, 0.625%, 1.25%, 2.5% and 5% of vapor saturation at 20°C). Each concentration was presented for only one block of 20 trials. As previously described in detail [e.g., [18,19]], the complex pattern of sniffing response to each stimulus was computer deconstructed into 14 response measures: sniff frequency; the number of inspiratory and expiratory sniffs; the duration, volume, average flow rate, and peak flow rate of an inspiratory and expiratory sniff; the total inspiratory and expiratory volume; and the total apneic duration.

### Data analysis

As noted above, sniffing patterns can be deconstructed into a relatively large number of variables (e.g., 14). In this respect, the multivariate nature of the data does not permit a direct evaluation of experimental main effects. Further, evaluation of any single variable is not sufficient to assess the import of the response to odorant stimuli (*ibid*). Consequently, following our previously established method, we took an approach that is standard in the field of psychology, namely, to create an "index" that incorporates the animal's behavioral responses into a single principal measure [4,18-21]. Using this approach, we have shown that gestational ethanol exposure tunes the behavioral olfactory responses to ethanol odor in infant rats (P15) [19,20]. This effect persists into adolescence (P28–P42) [4,21]. Moreover, re-exposure in adolescence augments the fetal exposure effects [4]. Importantly, Youngentob and Glendinning [20] demonstrated that fetal ethanol induced increases in the value of the "index" predicted enhanced ethanol avidity in the same animal. Moreover, the elevated intake of ethanol was causally linked to the enhanced odor response.

Briefly, principal components analysis (PCA) was first used to reduce the 14 dimensions (i.e. variables) of each hypothesis specific data set to two uncorrelated dimensions (Factors 1 and 2) (Note that, for each explicit hypothesis of this study there was a specific relevant effect to be evaluated such that what was relevant to evaluate one hypothesis was not most relevant to test another.) The values of the two resultant PCA factors, in turn, defined the animals' behavioral response at each of the five stimulus concentrations presented. In short, the PCA analysis was used to reduce the data set for each animal from a 14 × 5 to a 2 × 5 data matrix.

To construct a hypothesis specific two-dimensional composite reflexive sniffing index for each animal that summarized the sniffing response across all concentrations tested the following procedure was used. For each PCA factor we separately estimated the coefficient for each of the five stimulus response measures (one for each concentration tested) by performing a multivariate analysis with the five odorant-induced behavioral response measures as the dependent variables and treatment as the independent variable. For each animal the final composite index value for each factor was the summation of the constant from the regression analysis plus the animal's respective factor value at each concentration of odorant tested times its' respective estimated coefficient. Thus, each animal's 2 × 5 data matrix was reduced to a pair of X and Y coordinates that represented the relative physical location of an animal in behavioral response space. These data were used in subsequent analyses.

In this study we focused on a specific set of *a priori *hypotheses, which using appropriate error terms, permitted us to make inferences of a causal nature. For each hypothesis, a multivariate analysis of variance (MANOVA) tested the 2-dimensional response index as a function the specific effects under consideration. Moreover, single degree of freedom *t-*test's were used to perform subsequent exploratory analyses.

## Results

### Adolescent behavior

**Figure 1:**
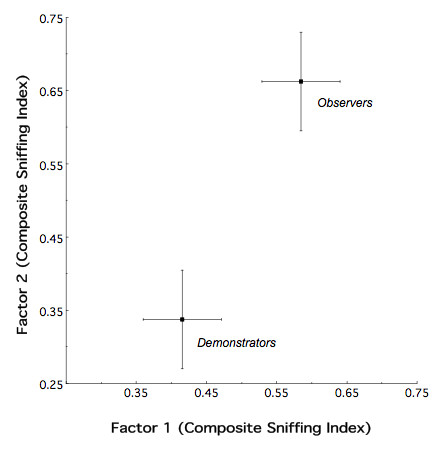
**Composite Sniffing Index for P37 observers and demonstrators that had been exposed to ethanol in adolescence**. Observer and demonstrator animals were found to respond differently to ethanol odor following adolescent exposure to the drug.

The primary goal of the adolescent evaluation was to determine whether, and to what degree, naïve ethanol observer and demonstrator animals differentially respond to ethanol odor as a consequence of their ethanol-related experience. Figure 1 illustrates the mean (± 2-dimensional s.e.m.) relative position of the observer and demonstrator animals in a stimulus-induced behavioral response space. MANOVA demonstrated an overall significant effect of mode of ethanol exposure (i.e. observer vs. demonstrator) on the reflexive sniffing response to ethanol odor at P37 (*F*_2,14 _= 5.75, *p *< 0.02). There was no evidence of a sex effect (*F*_2,14 _= 1.83, *p *> 0.15) or sex by treatment interaction (*F*_2,14 _= 0.56, *p *> 0.55).

The test of the above hypothesis was predicated on our previous finding that adolescent observer rats exposed to ethanol odor through social interaction with an intoxicated peer resulted in an enhanced olfactory response (to ethanol odor) relative to observer animals exposed to a water demonstrator [4]. The prior result not withstanding, in order to fully interpret the meaning of the current finding it was necessary to consider: (1) whether, relative to i.g. infused water controls, i.g. ethanol exposure, itself, results in an enhanced olfactory response; and (2) if so, to what extent the magnitude of the effect differs from that of ethanol vs. water observers.

In consideration of question 1, above, we conducted separate exploratory analyses of ethanol observers and demonstrators. Relative to their respective water controls, we tested the extent to which each form of exposure resulted in an enhanced ethanol odor response. MANOVA demonstrated that, in keeping with our previous study [4], ethanol observers significantly differed from controls (*F*_2,14 _= 6.30, nominal *p *< 0.015). Moreover, ethanol demonstrator animals also significantly differed from control exposure (*F*_2,14 _= 7.37, nominal *p *< 0.01). For both modes of exposure there was no evidence of an overall effect of sex (observers: *F*_2,14 _= 2.38, nominal *p *> 0.10; demonstrators: *F*_2,14 _= 1.08, nominal *p *> 0.35) or sex by treatment interaction (observers: *F*_2,14 _= 0.49, nominal *p *> 0.60; demonstrators: *F*_2,14 _= 1.37, nominal *p *> 0.25). In short, these findings confirmed our expectation that while ethanol observer and demonstrator animals differed in their response to ethanol odor, both modes of exposure yielded an enhanced response relative to control exposure.

**Figure 2:**
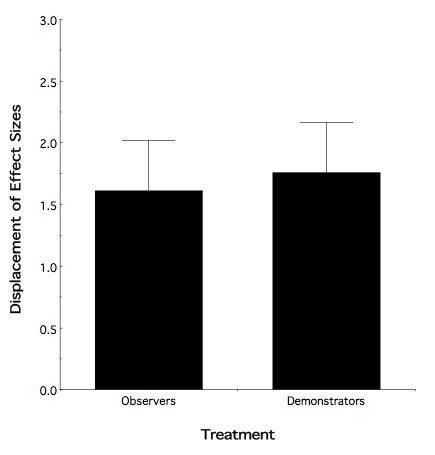
**Displacement of Effect Sizes at P37 as a function of mode of adolescent treatment**. Both modes of ethanol exposure led to similar magnitudes of behavioral enhancement as compared to their respective water controls.

Given the above result, we determined the magnitude of the differential effect between each treatment group and their respective control (i.e. question 2, above). To accomplish this, we calculated a displacement of effect sizes between the mean locations, in two dimensions, of each specific mode of adolescent exposure relative to its water control. That is, for each dimension we first divided the magnitude of the difference by the standard deviation and then calculated the displacement vector. This resulted in a single-dimensional value for each mode of exposure that represented the magnitude of treatment vs. control effect. Interestingly, as illustrated in Figure 2, the magnitude of the behavioral alteration, relative to their respective controls, was the same for observers and demonstrators (two-tailed *t *[15] = -0.248; nominal *p *> 0.50).

The foregoing results implied that observer and demonstrator animals that experienced water exposure did not respond equivalently to ethanol odor. That is, to achieve the results illustrated in Figure 2 water demonstrators would have to respond with sniffing index values significantly lower than those of water observers. Therefore, we explored whether the mode of water exposure (i.e. observer vs. demonstrator) had a differential effect on the behavioral response to ethanol odor. MANOVA demonstrated a significant effect of mode of water exposure (*F*_2,14 _= 7.19, nominal *p *< 0.01) suggesting, perhaps, an untoward consequence of the intubation procedure. There was no evidence of a sex (*F*_2,14 _= 0.03, nominal *p *> 0.95) or sex by treatment interaction (*F*_2,14 _= 0.16, nominal *p *> 0.85).

### Adult behavior

**Figure 3:**
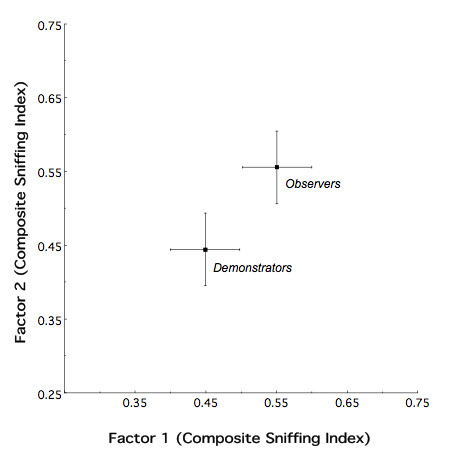
**Composite Sniffing Index for P90 observers and demonstrators that had been exposed to ethanol in adolescence**. Observer and demonstrator animals were found to respond similarly to ethanol odor in adulthood, following prior adolescent exposure to the drug.

In keeping with the above, the goal of the analysis was to determine: (1) whether adult, ethanol observer and demonstrator animals, exposed as adolescents, differ in their response to ethanol odor; and (2) whether, and to what degree, either or both modes of exposure differ from their respective water controls. Regarding the first question, MANOVA provided no strong evidence of an overall effect of mode of exposure on the behavioral response to ethanol odor at P90 (*F*_2,14 _= 2.77 *p *> 0.10) (Figure 3). Further, there was no evidence of an effect of sex (*F*_2,14 _= 0.26, *p *> 0.77) or sex by treatment interaction (*F*_2,14 _= 1.35, *p *> 0.28).

With respect to the second question, however, ethanol observer (*F*_2,14 _= 13.12, nominal *p *< 0.001) and demonstrator (*F*_2,14 _= 22.59, nominal *p *< 0.00005) animals both significantly differed from their individual water controls, indicating a persistence of the enhanced ethanol odor response into adulthood. There was no evidence of an overall effect of sex for either mode of exposure (observers: *F*_2,14 _= 0.27, nominal *p *> 0.81; demonstrators: *F*_2,14 _= 0.51, nominal *p *> 0.61). We observed a significant sex by treatment interaction in observer animals only (observers: *F*_2,14 _= 4.84, nominal *p *< 0.03; demonstrators: *F*_2,14 _= 1.80, nominal *p *> 0.20).

**Figure 4:**
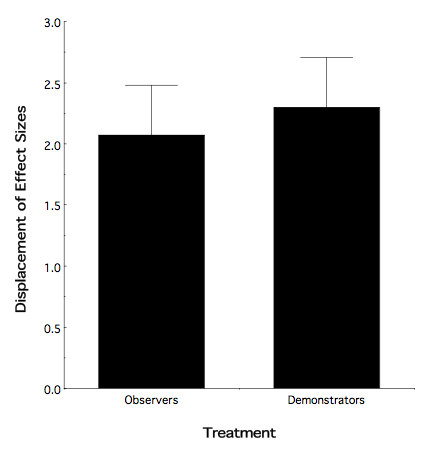
**Displacement of Effect Sizes at P90 as a function of mode of adolescent treatment**. Both modes of adolescent ethanol exposure resulted in persistence of the behavioral effect into adulthood (see text for details).

To further dissect these findings, we again calculated a displacement of effect sizes between ethanol-exposed animals relative to their water controls. As seen in Figure 4, ethanol observers and demonstrators both displayed a similar magnitude of alteration as compared to their water controls (two-tailed *t *[15] = -0.393; nominal *p *> 0.50). However, the interaction noted above suggested a differential effect in male and female observers. Interestingly, the female ethanol observers are driving the lion's share of the overall finding of an enhanced behavioral response to ethanol odor in observer animals at P90 (displacement of effect sizes: Females = 3.16; Males = 1.47).

## Discussion

Using the social transmission of food odor preference paradigm originally described by Galef and colleagues [1,3], several studies have demonstrated that both naïve infant and adolescent observer rats will increase their preference for ethanol odor, as well as manifest enhanced ethanol intake, as a consequence of social interaction with an ethanol intoxicated peer [e.g., [5-9]]. The underlying mechanism for this outcome is the transmission of food preference information through olfactory cues. Given that blood born odorants stimulate olfaction [11-13] and acute intoxication via i.g. infusion has been shown to yield altered ethanol odor responses (albeit aversion) [14-16], it is noteworthy that ethanol-related studies using social transmission have fundamentally ignored the odor-guided responses of the demonstrator. Especially so given the knowledge that human adolescents can experience ethanol either directly, through ingesting the drug, or indirectly, by interacting with an intoxicated peer in a social setting.

The present study extends upon prior findings by investigating whether and to what degree adolescent ethanol observer and demonstrator animals differ in their response to ethanol odor. Moreover, we examined the extent to which either or both forms of ethanol experience yield persistence of any behavioral odor-mediated effect into adulthood.

Here, we report that: (1) both adolescent observers and demonstrators show an enhanced response to ethanol odor; (2) nonetheless, observer and demonstrator animals significantly differ in their response to ethanol odor (Figure 1); and (3) although the enhanced odor response to ethanol was greater in observers, the magnitude of the enhanced behavioral change, relative to their respective controls, was equivalent for both modes of exposure (Figure 2). The foundation for this latter observation was the finding that water observers and demonstrators differed in their stimulus-induced sniffing response to ethanol odor, with the control demonstrator animals having a relatively reduced reflexive response as compared to control observers. The mechanism for this relative shift in demonstrator animals (both ethanol and water) as compared to observers is unknown at this time. However, one potential explanation is the method of demonstrator exposure, namely, intragastric infusion. Observers received odor exposure via social interaction. By contrast, ethanol demonstrators likely experienced hematogenic odor exposure, the post-ingestive consequences of ethanol, and what we suggest is the stress of intragastric infusion. With this in mind, recall that previous findings have shown that exposure-induced increases in ethanol avidity are causally linked to the enhanced odor response using our behavioral measure of odor responsivity [20]. Thus, the differential results for the water observers and demonstrators in the present study suggest the possibility that a potential untoward effect of intragastric infusion, itself, devalued the enhancing effects of the ethanol-related experience in ethanol demonstrators. Indeed, adolescents are more sensitive to stressors with exposure, in some instances, producing long lasting effects [22]. Further, exposure to a stressor in adolescence has been shown to impact adolescent responses to ethanol, for example, decreasing ethanol intake [23]. Consequently, it is not unreasonable to suggest that the intubation procedure experienced by ethanol demonstrators may have lead to an alteration in the adolescent behavioral response to ethanol odor: which manifested itself as a devaluation of the ethanol exposure induced behavioral response index. In a similar fashion, ethanol odor testing may have represented an additional stressor to water demonstrators already sensitized by the intubation procedure: thereby resulting in diminished odor responsivity relative to water observers. Thus, future studies of the consequences of observer vs. demonstrator effects should consider other forms of more "natural" exposure. For example, having demonstrator animals self-administer a given aliquot of fluid would have an obvious distinct advantage over intragastric infusion.

Our finding of an enhanced ethanol odor response in adolescent and adult demonstrator animals stands in contrast to previous work indicating odor aversion following acute ethanol intoxication via intragastric infusion [14-16]. However, several important distinctions in study execution must be noted. In the present study, we examined animals (both adolescents and adults) exposed in adolescence within the context of a social transmission paradigm. By distinction, previous studies [14-16] were performed in pre-weanling animals, outside the context of a social setting. In other words, these previous studies investigated isolated, acute ethanol exposures. Moreover, several of these studies used ethanol doses approximately twofold higher then in the present experiment. Whether one or more of these variables are critical to the differential finding is unknown. Nonetheless, the present study extends upon this previous work by unambiguously demonstrating a set of relevant circumstances in which acute ethanol intoxication results in an enhanced behavioral response to ethanol odor rather than aversive.

Unlike the differential behavioral response to ethanol odor found between adolescent ethanol observers and demonstrators, no such differential effect was manifest in adulthood (Figure 3). However, both modes of ethanol exposure still significantly differed from their respective controls (Figure 4) demonstrating a clear-cut persistence of the adolescent exposure effect into adulthood. In this respect, it is tempting to suggest on the basis of Figure 4 that, on average, compared to Figure 2, the absolute behavioral consequence of adolescent ethanol exposure had become "larger". However, there is a caveat to such an interpretation. Recall that, Figures 2 and 4 represent displacement of effect sizes between exposed and control animals. As such, a change in the magnitude of the displacements could be a consequence of a shift in the ethanol odor response of either the exposed or control animals, or both. At present we cannot discern between these possible alternatives.

## Conclusion

The key findings of this study demonstrate that *de novo *adolescent ethanol exposure through either social interaction with an intoxicated peer or direct experience with the drug both yield an enhanced behavioral response to ethanol odor in adolescence as well as a persistence of these alterations into adulthood. Within the context of the social transmission paradigm the demonstrator results stand in contrast to previous studies of acute intoxication that demonstrated ethanol odor aversion. These findings have significant implications for our understanding of factors contributing to the progressive pattern of alcohol abuse. Epidemiological studies demonstrate: (a) a predictive relationship between prenatal ethanol exposure and the increased risk for adolescent ethanol abuse [24-28]; and (b) that postnatal experience increases the probability of long-term abuse [27,28]. The chemosensory attributes of ethanol (i.e. smell, taste and somatosensory) are thought to be important determinants of ethanol acceptance [29] and recent findings have shown that fetal exposure-induced enhancements in the behavioral response to ethanol odor directly contribute to increases in ethanol avidity [20]. Moreover, adolescent ethanol odor re-exposure through social interaction with an intoxicated peer augments the odor-guided behavior response of fetal exposure [4]. The current results further our understanding of this progressive human epidemiology by highlighting the consequences of two socially relevant modes of adolescent ethanol odor experience.

## Competing interests

The authors declare that they have no competing interests.

## Authors' contributions

Both authors were involved in the design of the study, interpretation of the data, and preparation of the manuscript; AME executed the study and analyzed the data. Both authors read and approved the final manuscript.

## References

[B1] Galef BG, Aslin RN, Alberts JR, Petersen MR (1981). Development of flavor preferences in man and animals: The role of social and nonsocial factors. Development of Perception Psychobiological Perspectives.

[B2] Galef BG, Stein M (1985). Demonstrator influence on observer diet preference: Analysis of critical social interactions and olfactory signals. Anim Learn Behav.

[B3] Galef BG, Wigmore SW (1983). Transfer of information concerning distant foods: A laboratory investigation of the 'information-centre' hypothesis. Anim Behav.

[B4] Eade AM, Sheehe PR, Molina JC, Spear NE, Youngentob LM, Youngentob SL (2009). The consequence of fetal ethanol exposure and adolescent odor re-exposure on the response to ethanol odor in adolescent and adult rats. Behav Brain Funct.

[B5] Hunt PS, Hallmark RA (2001). Increases in ethanol ingestion by young rats following interaction with intoxicated siblings: A review. Integr Physiol Behav Sci.

[B6] Hunt PS, Holloway JL, Scordalakes EM (2001). Social interaction with an intoxicated sibling can result in increased intake of ethanol by periadolescent rats. Dev Psychobiol.

[B7] Hunt PS, Lant GM, Carroll CA (2000). Enhanced intake of ethanol in preweanling rats following interactions with intoxicated siblings. Dev Psychobiol.

[B8] Fernandez-Vidal JM, Molina JC (2004). Socially mediated alcohol preferences in adolescent rats following interactions with an intoxicated peer. Pharmacol Biochem Behav.

[B9] Chotro MG, Kraebel KS, McKinzie DL, Molina JC, Spear N (1996). Prenatal and postnatal ethanol exposure influences preweanling rats' behavioral and autonomic responding to ethanol odor. Alcohol.

[B10] Honey PL, Galef BG (2003). Ethanol consumption by rat dams during gestation, lactation and weaning increases ethanol consumption by their adolescent young. Dev Psychobiol.

[B11] Van Dishoeck HAE, Versteeg N (1957). On the problem of hematogenic olfaction. Arch Otolaryngol.

[B12] Maruniak JA, Mason JR, Kostelc JG (1983). Conditioned aversions to an intravascular odorant. Physiol Behav.

[B13] Maruniak JA, Silver WL, Moulton DG (1983). Olfactory receptors respond to bloodborne odorants. Brain Res.

[B14] Molina JC, Chotro MG (1989). Acute alcohol intoxication paired with appetitive reinforcement: effects upon ethanol intake in infant rats. Behav Neural Biol.

[B15] Molina JC, Chotro MG, Spear NE (1989). Early (preweanling) recognition of alcohol's orosensory cues resulting from acute ethanol intoxication. Behav Neural Biol.

[B16] Hunt PS, Molina JC, Rajachandran L, Spear LP, Spear NE (1993). Chronic administration of alcohol in the developing rat: expression of functional tolerance and alcohol olfactory aversions. Behav Neural Biol.

[B17] Varlinskaya EI, Spear LP, Spear NE (2001). Acute effects of ethanol on behavior of adolescent rats: Role of social context. Alcohol Clin Exp Res.

[B18] Youngentob SL (2005). A method for the rapid automated assessment of olfactory function. Chem Senses.

[B19] Youngentob SL, Kent PF, Sheehe PR, Molina JC, Spear NE, Youngentob LM (2007). Experience-induced fetal plasticity: The effect of gestational ethanol exposure on the behavioral and neurophysiologic olfactory response to ethanol odor in early postnatal and adult rats. Behav Neurosc.

[B20] Youngentob SL, Glendinning JI (2009). Fetal ethanol exposure increases ethanol intake by making it smell and taste better. Proc Natl Acad Sci USA.

[B21] Middleton FA, Carrierfenster K, Mooney SM, Youngentob SL (2009). Gestational ethanol exposure alters the behavioral response to ethanol odor and the expression of neurotransmission genes in the olfactory bulb of adolescent rats. Brain Res.

[B22] Spear LP (2000). The adolescent brain and age-related behavioral manifestations. Neurosci Biobehav Rev.

[B23] Brunell SC, Spear LP (2005). Effects of stress on the voluntary intake of a sweetened ethanol solution in pair-housed adolescent and adult rats. Alcohol Clin Exp Res.

[B24] Alati R, Al Mamum A, Williams GM, O'Callagham M, Najman JM, Bor W (2006). In utero alcohol exposure and prediction of alcohol disorders in early adulthood: A birth cohort study. Arch Gen Psychiatry.

[B25] Baer JS, Barr HM, Bookstein FL, Sampson PD, Streissguth AP (1998). Prenatal alcohol exposure and family history of alcoholism in the etiology of adolescent alcohol problems. J Stud Alcohol.

[B26] Baer JS, Sampson PD, Barr HM, Connor PD, Streissguth AP (2003). A 21-year longitudinal analysis of the effects of prenatal alcohol exposure on young adult drinking. Arch Gen Psychiatry.

[B27] Streissguth A Teratogenic and genetic influences on adolescent and adult alcohol use and abuse. Symposium presented at the annual meeting of the Research Society on Alcoholism, Hilton Head, SC; June 1998.

[B28] Yates WR, Cadoret RJ, Troughton EP, Stewart M, Giunta TS (1998). Effect of fetal alcohol exposure on adult symptoms of nicotine, alcohol, and drug dependence. Alcohol Clin Exp Res.

[B29] Bachmanov AA, Kiefer SW, Molina JC, Tordoff MG, Duffy VB, Bartoshuk LM, Mennella JA (2003). Chemosensory factors influencing alcohol perception preferences and consumption. Alcohol Clin Exp Res.

